# 经支气管内超声引导针吸活检术在小细胞肺癌与非小细胞肺癌诊断中的应用价值

**DOI:** 10.3779/j.issn.1009-3419.2020.101.02

**Published:** 2020-06-20

**Authors:** 晓刚 谭, 宝东 刘, 若天 王, 毅 张

**Affiliations:** 100053 北京，首都医科大学宣武医院胸外科 Department of Thoracic Surgery, Xuan Wu Hospital of Capital Medical University, Beijing 100053, China

**Keywords:** 经支气管内超声引导针吸活检术, 小细胞肺癌, 肺肿瘤, 诊断准确率, Endobronchial ultrasound guided transbronchial needle aspiration, Small cell lung cancer, Lung neoplasms, Diagnosis accuracy

## Abstract

**背景与目的:**

纤维支气管镜腔内超声引导针吸活检术（endobronchial ultrasound guided transbronchial needle aspiration, EBUS-TBNA）作为近年来发展的新技术，具有操作简单、创伤小、准确率和安全性高及可重复性等优点，已成为进行肺癌诊断和纵隔分期的新标准。由于小细胞肺癌（small cell lung cancer, SCLC）和非小细胞肺癌（non-small cell lung cancer, NSCLC）的生物学特性以及治疗方案的不同，因此，在早期能诊断并鉴别的病理类型，对于肺癌的分期、治疗及预后均有很重要意义。本文探讨EBUS-TBNA在诊断SCLC和NSCLC中的准确率及敏感性。

**方法:**

回顾性分析2012年1月-2018年12月首都医科大学宣武医院胸外科进行142例经支气管内超声引导针吸活检术患者的临床资料，选取最终确诊85例SCLC和NSCLC患者，比较两组差异。

**结果:**

最终经免疫组化病理明确SCLC 45例，其中经EBUS-TBNA明确诊断为SCLC 42例，诊断准确率、敏感度分别为93.3%（42/45）、100.0%（42/42）。经细胞学检查明确诊断22例，诊断准确率为48.9%（22/45）；经病理明确诊断NSCLC 40例，其中经EBUS-TBNA明确诊断为NSCLC 35例，诊断准确率、敏感度分别为87.5%（35/40）、100.0%（35/35）。经细胞学检查明确诊断11例，诊断准确率为27.5%（11/40）；EBUS-TBNA在SCLC组的诊断准确率明显高于NSCLC组，且有统计学意义（*P* < 0.05）。

**结论:**

EBUS-TBNA用于诊断SCLC较NSCLC的准确率高。EBUS-TBNA作为微创技术，可协助SCLC早期诊断，及时治疗。

## Abstract (en)

**Background and objective:**

As a new technique developed in recent years, endobronchial ultrasound guided transbronchial needle aspiration (EBUS-TBNA) has the advantages of simple operation, minimal invasive, high accuracy, safety and repeatability. It has become a new standard for lung cancer diagnosis and mediastinal staging. Because small cell lung cancer (SCLC) and non-small cell lung cancer (NSCLC) have different biological characteristics and treatment methods, it is very important to diagnose and differentiate the types of lung cancer in the early stage of lung cancer for the staging, treatment and prognosis of lung cancer. This article evaluated the accuracy and sensitivity of EBUS-TBNA in the diagnosis of SCLC and NSCLC.

**Methods:**

From January 2012 to December 2018, the clinical data of 85 patients with SCLC and NSCLC who performed EBUS-TBNA in Xuan Wu Hospital CMU were retrospectively analyzed and the differences between the two groups were compared.

**Results:**

45 cases of SCLC were confirmed by immunohistochemistry and pathology. 42 cases of SCLC were diagnosed by EBUS-TBNA. The accuracy and sensitivity of diagnosis were 93.3% (42/45) and 100.0% (42/42), respectively. The positive rate of diagnosis was 48.9% (22/45) in 22 cases diagnosed by cytology, and 40 cases diagnosed by pathology, including 35 cases diagnosed by EBUS-TBNA. The accuracy and sensitivity of diagnosis were 87.5% (35/40) and 100.0% (35/35), respectively. The positive rate of diagnosis was 27.5% (11/40) in 11 cases diagnosed by cytology. The diagnostic sensitivity of EBUS-TBNA in SCLC group was significantly higher than that in NSCLC group (*P* < 0.05).

**Conclusion:**

EBUS-TBNA is more sensitive in the diagnosis of SCLC than NSCLC. As a minimally invasive technique, EBUS-TBNA can assist SCLC in early diagnosis and timely treatment.

纤维支气管镜腔内超声引导针吸活检术（endobronchial ultrasound guided transbronchial needle aspiration, EBUS-TBNA）作为近年来发展的新技术，通过实时超声引导下行支气管针吸活检，具有操作简单、创伤小、准确率和安全性高及可重复性等优点，具有重要的临床价值和广泛的应用前景，已成为进行肺癌诊断和纵隔分期的新标准^[1]^。

肺癌根据生物学特性，可分为非小细胞肺癌（non-small cell lung cancer, NSCLC）和小细胞肺癌（small cell lung cancer, SCLC），NSCLC发病率为85%-90%，与SCLC相比NSCLC癌细胞生长分裂较慢，扩散转移相对较晚，目前采用手术联合化疗的方式进行治疗。SCLC发病率为10%-15%，男性多发于女性，发病部位以大支气管（中心型）居多，细胞分化快，易形成较大体积的肿瘤，甚至会蔓延至淋巴结或全身其他器官，对放化疗敏感，故SCLC的治疗应以全身化疗为主，联合放疗和手术为主要治疗手段^[2]^。由于NSCLC与SCLC的生物学特性以及治疗方案的不同，因此，在早期能诊断并鉴别的病理学类型，对于肺癌的分期、治疗及预后均有很重要意义。本文通过回顾性分析2012年1月-2018年12月首都医科大学宣武医院胸外科进行142例经支气管内超声引导针吸活检术患者的临床资料，选取最终确诊85例SCLC和NSCLC患者，比较经支气管内超声引导针吸活检术在SCLC与NSCLC诊断中的准确率及敏感性。

## 1 资料与方法

### 1.1 临床资料

回顾性分析2012年1月-2018年12月于首都医科大学宣武医院手术室行EBUS-TBNA检查的所有患者。纳入标准：①术前行胸部计算机断层扫描（computed tomography, CT）显示纵隔/肺门病灶（包括短径 > 1 cm的淋巴结及肿块）；②综合患者病史及辅助检查等结果分析后高度疑诊原发性肺癌；③术前完善心电图、凝血功能等检查排除超声支气管镜检查相关禁忌证；④术前告知患者及患者家属手术操作过程及相关风险并签署手术同意书；⑤经支气管内超声引导针吸活检术或其他外科方法最终确诊SCLC和NSCLC的患者。排除标准：①可通过常规支气管镜或体表肿大淋巴结穿刺活检等手段明确诊断者；②存在严重心、肺功能衰竭等手术禁忌者；③不能获取完整临床资料者。此次研究共纳入85例患者，SCLC 45例，NSCLC 40例。纵隔淋巴结的命名根据国际肺癌研究协会（International Association for the Study of Lung Cancer, IASLC）制定的肺癌区域淋巴结分布图谱（2009）^[3]^。

### 1.2 EBUS-TBNA操作过程

操作前常规行2%利多卡因10 mL雾化吸入15 min-20 min。操作时，患者取去枕仰卧位，行静脉全身麻醉（保留自主呼吸），经5号内镜面罩连接麻醉机吸氧（5 L/min）。如患者出现舌后坠则适当托起下颌，随患者自主呼吸手法辅助通气。监测患者的心率、血压及脉搏血氧饱和度。首先进行常规支气管镜检查，并彻底清理气道内分泌物，以减少对后续检查的干扰。而后，经口置入超声支气管镜（BF-UC260F-OL8; Olympus, Japan），利用超声图像顺序探查纵隔内各站淋巴结，对于影像学肿大或可疑转移淋巴结（> 5 mm）均进行穿刺活检。明确目标淋巴结及气管壁穿刺部位（软骨环间隙）后，经工作通道置入EBUS-TBNA专用的22 G穿刺活检针（NA-201SX-4022; Olympus, Japan），在超声图像的实时监视下进行穿刺活检。穿刺前常规进行多普勒检查，以避免损伤血管。穿刺标本分别经涂片、固定（95%乙醇）及染色后进行细胞病理学检查；所获得的组织标本经4%甲醛固定后送病理科检查。如需对多站淋巴结进行穿刺，为避免交叉污染，需更换穿刺活检针。

### 1.3 结果判断

细胞学或组织病理学和免疫组织化学任何一种检查方法找到SCLC或NSCLC恶性肿瘤细胞即判断为阳性，未找到上述细胞则视为阴性。若EBUS-TBNA术后病理诊断结果为阴性，但患者术后行经皮肺穿刺活检、胸腔镜、纵隔镜、开胸手术等有创操作获得明确的病理诊断，则以后者的病理结果为最终诊断；若EBUS-TBNA术后病例诊断结果为阴性，且患者因各种因素未能进一步行有创检查，根据临床诊断行经验性治疗，密切随访至少半年临床诊断得到验证后，以患者的临床诊断为最终诊断。

### 1.4 统计学处理

应用SPSS 13.0统计学软件。计数资料以百分率（%）表示，组间比较采用*χ*^2^检验。以*P* < 0.05为差异有统计学意义。

## 2 结果

### 2.1 一般资料

45例SCLC患者中，男性34例，女性11例；年龄45岁-86岁，中位年龄为61岁；33例有吸烟史，吸烟指数90-4600，中位值为600。发射单光子计算机断层扫描（emission computed tomography, ECT）胸部肿瘤代谢：T/N=1.4-6.69，中位值: 3.6。正电子发射型计算机断层显像（positron emission computed tomography, PET）-计算机断层扫描（computed tomography, CT）病灶标准摄取值（standard uptake value, SUV）4.0-42，中位SUV值为16.0。除1例肿瘤标志物神经元特异性烯醇化酶（neuron-specific enolase, NSE）（我院正常值：0 µg/L-17 µg/L）和胃泌素释放肽前体（pro-gastrin-releasing peptide, Pro-GRP）（我院正常值：0 µg/L-69 µg/L）同时为阴性外，其余病例至少有1项高于正常值。40例NSCLC患者中，男性36例，女性4例；年龄37岁-80岁，中位年龄为61岁；31例有吸烟史，吸烟指数400-2400，中位值为800。ECT胸部肿瘤代谢：T/N=2.2-6.96，中位值：3.2。PET-CT SUV值4.0-7.94，中位SUV值为5.9。癌胚抗原（carcinoembryonic antigen, CEA）或细胞角蛋白片段（Cyfra21-1）至少有1项高于正常值。两组患者在性别、年龄等基本情况方面差异无统计学意义（*P* > 0.05），具有可比性。

### 2.2 穿刺情况

最常穿刺部位淋巴结是7组，其次是4R组。同一淋巴结穿刺针数1针-3针，中位穿刺数2针；同一患者淋巴结穿刺个数1个-2个，中位穿刺数1个。SCLC全组共穿刺57组淋巴结，其中2R组1例，4R组20例，4L组2例，5组3例，7组25例，10R组3例，10L组3例。NSCLC全组共穿刺48组淋巴结，其中2R组1例，4R组18例，4L组1例，5组8例，7组18例，10R组1例，10L组1例（表 2）。穿刺过程中均未发生纵隔大血管破裂出血、气胸、纵隔气肿等并发症。

**2:** 病理诊断 Pathology diagnosis

Pathology diagnosis	*n*	Diagnosed by EBUS	False diagnosis by EBUS	Accuracy (%)
SCLC by IHC	45	42	3	93.3
NSCLC by IHC	40	35	5	87.5
TOTAL	85	77	8	90.6
NSCLC by cytology	45	22	23	48.9
NSCLC by cytology	40	11	29	27.5
Total	85	33	42	38.8
IHC: immunohistochemistry; EBUS: endobronchial ultrasound.				

### 2.3 诊断结果

最终经免疫组化病理明确SCLC 45例，其中经EBUS-TBNA明确诊断为SCLC 42例，诊断正确率、敏感度分别为93.3%（42/45）、100.0%（42/42）。经病理明确诊断NSCLC 40例，其中经EBUS-TBNA明确诊断为NSCLC 35例，诊断正确率、敏感度分别为87.5%（35/40）、100.0%（35/35）。EBUS-TBNA在SCLC组的诊断敏感度明显高于NSCLC组，且差异具有统计学意义（*P* < 0.05）。全部患者均常规行细胞学和组织病理学检查（HE染色）。45例SCLC患者经细胞学检查明确诊断22例，诊断准确率为48.9%（22/45）；40例NSCLC患者经细胞学检查明确诊断11例，诊断准确率为27.5%（11/40）。

**1:** 穿刺部位及诊断率 Mediastinal lymph node stations and diagnosis accuracy by stations

Lymph node stations	Total	Positive	Negative	Accuracy (%)
SCLC				
2R	1	0	1	0.0
4R	20	19	1	95.0
4L	2	2	0	100.0
5	3	2	2	66.7
7	25	25	0	100.0
10R	3	3	0	100.0
10L	3	2	1	66.7
Total	57	53	4	93.0
NSCLC				
2R	1	1	0	100
4R	18	16	2	88.9
4L	1	1	0	100
5	8	5	3	62.5
7	18	18	0	100
10R	1	1	0	100
10L	1	1	0	100
Total	48	43	5	89.6
Total	105	96	9	91.4
NSCLC: non-small cell lung cancer; SCLC: small cell lung cancer.				

## 3 讨论

手术是现阶段NSCLC预后最好的治疗方式，纵隔淋巴结的评估是患者能否接受手术治疗的决定性因素之一，直接关系到治疗策略的制定，分期的准确性显得尤为重要。SCLC是一种高度恶性的肿瘤，属于肺癌的未分化类型，其增殖速度快，恶性程度高，转移早，患者5年生存率低^[6, 7]^。因此，早发现、早诊断、早治疗是防治SCLC的关键。美国胸科医师协会在2013年临床实践指南^[8]^中建议，应采用最简单、最安全的方法诊断SCLC。Harrow等^[9]^发现，在相同大小的淋巴结中，经气管镜穿刺针吸活检（transbronchial needle aspiration, TBNA）诊断SCLC和NSCLC的准确率分别为62%和48%。但EBUS-TBNA在SCLC和NSCLC诊断中的准确率及敏感性比较却鲜有报道。本文回顾性分析2012年1月-2018年12月首都医科大学宣武医院胸外科进行142例经支气管内超声引导针吸活检术患者的临床资料，最终确诊85例SCLC和NSCLC患者，比较EBUS-TBNA在SCLC和NSCLC诊断中的准确率。结果经EBUS-TBNA明确诊断为SCLC 42例，诊断准确率为93.3%（42/45）。经病理明确诊断NSCLC 40例，其中经EBUS-TBNA明确诊断为NSCLC 35例，诊断准确率为87.5%。EBUS-TBNA在SCLC组的诊断敏感度明显高于NSCLC组，且具有统计学意义。

关于EBUS-TBNA在SCLC早期诊断中作用的研究并不多。Wada等^[10]^的研究结果显示，EBUS-TBNA诊断SCLC的准确率及敏感性分别为96.4%、100%。Murakami等^[11]^的研究结果显示，EBUS-TBNA诊断SCLC的准确率为97%（97/100）。本研究结果显示，EBUS-TBNA诊断SCLC的准确率为93.3%（42/45），高于TBNA（64%-90.5%）^[9, 12, 13]^。文献^[14-16]^报道，EBUS-TBNA纵隔淋巴结分期的准确率为89%-99%，敏感性为100.0%，与纵隔镜相近。本组病例中，EBUS-TBNA在NSCLC中的准确率及敏感性分别为87.5%、100.0%，较文献报道略低，较SCLC敏感性也较低。分析这可能与SCLC的生物学侵袭性如大部分患者在初次诊断时已有肺门和纵隔淋巴结转移，和（或）SCLC细胞之间黏附力下降有关。同时，非小细胞未分化癌往往由于分化程度更低，免疫组化无典型表现增加了诊断的难度，故诊断率相对较低。

EBUS-TBNA在TBNA的基础上引入实时凸面超声内镜引导，开启多普勒血流检查，可以防止误穿血管，同时准确获取肺门肿块、纵隔的病变细胞和组织，极大地提高了TBNA的安全性和诊断率。但其在临床应用中也有一定的局限性，主要受取材部位和取材数量的影响。由于EBUS-TBNA吸引活检针孔径较小，取材数量有限，恶性肿瘤假阴性病例中，镜下仅有血凝块伴少量淋巴细胞，考虑与穿刺标本取材不足或纵隔淋巴结内可能出现微小转移灶、合并肉芽肿性炎、钙化等导致假阴性结果。另外，操作医师熟练程度的不同以及病灶的大小、位置的不同，也会导致假阴性结果，临床医师应充分考虑到。

Hermens等^[17]^推荐，在肺癌分期中每个病变部位穿刺3次可以得到最高诊断率。也有研究^[18, 19]^发现，尽管只有21号或22号穿刺针供选择，但仍有约75%的患者穿刺2针即可获得组织病理学标本。在本研究中，平均每个病变穿刺2针，SCLC得出93.3%（42/45）阳性率还是可以接受的。EBUS-TBNA在NSCLC中的准确率为87.5%，较文献报道略低，较小细胞准确率也较低。建议在NSCLC行EBUS-TBNA时，如果取材效果不满意，可以在保证安全前提下，重复取材。本研究EBUS-TBNA穿刺共出现8例假阴性患者，3例行CT引导下经皮肺穿刺，3例行气管镜，1例纵隔镜，1例胸腔镜手术证实病理。对于EBUS-TBNA阴性患者的处理，目前尚存在争议。Defranchi等^[20]^报道，EBUS-TBNA开展早期假阴性率高达28%。虽然大多数学者认为EBUS-TBNA阴性结果仍有必要进一步行外科手段确认^[21, 22]^，然而也有研究显示EBUS-TBNA联合纵隔镜检查的总体诊断率与单纯EBUS-TBNA比较，两者并无显著差异^[23]^。但8例同时有肿瘤标志物升高及肿瘤代谢值增高，提示恶性可能，建议有高危因素患者通过其他方法取得病理诊断。

此外，有研究结果提示不同穿刺部位的阳性率不同，其中主肺动脉窗淋巴结（N5组）穿刺阳性率相对较低，本研究7、4R、5组淋巴结穿刺频率最高，穿刺阳性率分别为100%（42/42）、92.1%（35/38）、63.6%（7/11），其中5组中有4例穿刺阴性淋巴结，SCLC中1例（1/3）和NSCLC中3例（3/8），占所有假阴性病例的50%（4/8）。考虑可能与上述部位的淋巴结穿刺相对困难、非常规穿刺取材部位（N5组淋巴结）^[24]^以及穿刺定位标志不明确相关。

近年来，国际肺癌研究协会对于肺癌的分型分类也进行了更新。在小标本诊断中，更需要应用免疫组化区分具体病理类型以指导治疗。因此，对于肺癌患者，取得活检组织不仅需要能够提供肿瘤的病理诊断，还需要提供具体分型的信息。Navavi等^[25]^报道了774例小标本患者进行免疫组化染色后进行分型，减少了NSCLC组织不明确型的诊断率，也提示了免疫组化对于肺癌分型的重要性。本研究中，单纯细胞学刷片仅48.9%（22/45）见SCLC细胞，NSCLC经细胞学检查明确诊断11例，诊断准确率为27.5%（11/40）；组织病理学检查的诊断阳性率显著高于细胞学检查。提示细胞学需与组织学检查联合，减少漏诊和不完全诊断。

大量的研究表明：EBUS-TBNA具有较高的安全性及较少的并发症。常见并发症有：穿刺部位少量出血、气道痉挛及低氧。仅少数患者术后发生气胸、纵隔气肿和纵隔出血等并发症，其发生率不足1%。因EBUS-TBNA是在超声实时监视下穿刺，误穿到大血管导致大出血的可能性很小，又因穿刺针为22 G细针，绝大多数患者仅在穿刺点有少许出血。本研究中85例患者主要并发症为穿刺点少量出血，均未进行特殊处理，未出现气胸、纵隔气肿和纵隔出血等较严重并发症。综上所述，EBUS-TBNA技术是一项方便、安全、微创的检查方法。EBUS-TBNA用于小细胞癌较NSCLC诊断的准确性较高。EBUS-TBNA作为微创技术，可协助SCLC早期诊断，及时治疗。有理由相信EBUS-TBNA在将来临床工作中必将发挥越来越重要的作用。
